# Husbands’ knowledge and involvement in sexual and reproductive health rights of women in Bahir Dar City, Northwest Ethiopia: a community-based study

**DOI:** 10.3389/fpubh.2024.1359756

**Published:** 2024-04-17

**Authors:** Mekdes Mengesha Jemberie, Meseret Zewdu, Bayew Kelkay Rade

**Affiliations:** ^1^Tibebe Gihon Specialized and Comprehensive Hospital, Bahir Dar University, Bahir Dar, Ethiopia; ^2^Department of Gender and Developmental Studies, Faculty of Social Science, Bahir Dar University, Bahir Dar, Ethiopia; ^3^Department of General Midwifery, School of Midwifery, College of Medicine and Health Sciences, University of Gondar, Gondar, Ethiopia

**Keywords:** involvement, sexual health, sexual rights, reproductive health, reproductive rights

## Abstract

**Background:**

Sexual and reproductive health rights (SRHRs) are integral elements of the rights of everyone to the highest attainable standard of physical and mental health, but they are the most underdeveloped and least understood sphere of rights, especially in Africa, including the country of Ethiopia. The implementation of women’s SRHRs is essential for achieving gender equality and promoting women’s rights. Husbands’ knowledge and involvement play a significant role in improving women’s practice of their SRHRs. However, there is limited information/data about the level of husbands’ knowledge and involvement in Northwest Ethiopia, including Bahir Dar City. Therefore, this study aimed to assess husbands’ knowledge, involvement, and factors influencing their involvement in women’s SRHRs.

**Methods:**

Community-based cross-sectional study design was conducted from March 20 to April 5, 2023, in Bahir Dar City, Northwest Ethiopia, among 391 husbands. Multi-stage sampling and simple random sampling technique were applied to select kebeles and study participants, respectively. Participants were interviewed face-to-face using structured and pretested questionnaire. Binary logistic regression was applied to identify associated factors, and a *p*-value of <0.05 was a cutoff point to declare statistical significance.

**Results:**

In this study, 50.6% (198/391) of the husbands had good knowledge about their wives’ SRHRs and 44.2% (173/391) (95% CI, 39.3–49.1%) of the husbands were involved when their wives practiced their SRHRs. Access training/education about sexual health [AOR = 5.99; 95% CI (2.7–13.2)], husbands’ advance educational level [AOR = 8.81; 95% CI (2.04–38)], good knowledge about SRHRs [AOR = 7.94; 95% CI (4.3–14.4)], low monthly income (<4,600 birr) [AOR = 9.25; 95% CI (4.2–20.5)], and had open discussion with family members and friends about SRHRs [AOR = 1.92; 95% CI (1.01–3.6)] were found to have significant association with husbands’ involvement.

**Conclusion:**

Husbands’ level of knowledge on SRHRs of women and their involvement remain low. Therefore, responsible concerned bodies need to work on the strategies that help to improve men involvement and knowledge, and tackle the above-mentioned factors influencing their involvement.

## Introduction

The global maternal mortality rate (MMR) in 2020 was estimated 223 per 1,000,000 live births, and sub-Saharan African region have reported extremely high MMR, which was over 1,000 (1). According to mini-2019-Ethiopian Demographic Health Survey (EDHS) report, MMR accounts 412 per 100,000 live births (2). This is because polices, programs, and strategies were not giving special attention to women and their roles for improving reproductive health rights; result high maternal morbidities and mortalities. Women sexual and reproductive rights need special attention since the violation occurred worldwide and have serious consequence on women’s health (3, 4).

Sexual and reproductive health rights (SRHRs) are defined as “these are the rights of sexual and reproductive health, that includes the right to freely control and responsibly decide on matters related to sexuality, including sexual and reproductive decision-making and the ability to seek sexual and reproductive healthcare; information, counseling, and care related to sexual function and satisfaction; prevention, detection, and management of sexual and gender-based violence and coercion; a choice of safe and effective contraceptive methods; safe and effective antenatal, childbirth and postnatal care; safe and effective abortion and post-abortion care services; prevention, management and treatment of infertility, sexual transmitted infections (STIs), including human immunodeficiency virus (HIV) infection; and prevention, screening, and treatment of reproductive cancers like cervical cancer” (5, 6). About 4.3 billion of reproductive age women globally have inadequate SRH services for their lives, and there is relatively low access in the low- and middle-income countries (LMICs) (7). In history, men focused almost exclusively on women’s fertility, and family planning program policies were implemented with limited male involvement (8). Evidence revealed that knowing husband’s understanding of his wife’s reproductive rights can aid in planning and intervening on maternal healthcare needs during pregnancy, childbirth, and afterward (9). Moreover, male involvement related to the practice of maternal healthcare service uptake and nutritional health has a positive association in reducing maternal and child morbidity and mortality (10). The large body of evidence said that women’s practice of their SRHRs is low without explaining men’s involvement because the source of data was only from women (11). In Nigeria, husbands’ involvement on safe motherhood was 26.3%-antenatal care (ANC), 19%-nutrition, and 4.6%-involved in family planning. Nearly, three-fourths (74.8%) of the husbands had poor knowledge of safe motherhood initiative practices (12). Husbands’ lack of knowledge on wives’ reproductive health right is the public health concern in Ghana, and 53.8% had poor knowledge about human rights and 66.7% denial of SRHRs services (13). The study carried out in Harar revealed that the level of husbands’ involvement in women SRHRs was 40.1% (14). Study among university students in Ethiopia also highlighted that 45.5% of them have no adequate knowledge on sexual and reproductive rights (9). Some literatures revealed that media exposure, high level of education, positive attitude, good knowledge and awareness, good sociocultural factors, and good policies play important roles in improving male involvement in women sexual and reproductive health rights (15–17). Generally, women are facing a challenge in exercising their SRHRs because they have less power in relationship due to their economic, political and sociocultural status that results gender disparities which strongly affect SRHRs of women (18). Husbands are the main household decision-makers in various cultures of many countries, including Ethiopia, which affect wives to exercise their reproductive health rights and cause morbidity and mortality of women (9, 13).

Husbands and wives in Ethiopia are openly communicating on different issues in life, but not focused on reproductive health rights (RHRs). Past studies determined women’s knowledge and their practice of SRHRs and reported that male dominance in women’s decision-making on sexual reproductive health rights (SRHRs) was proximal factor for poor practice of SRHRs. The implementation of proper and effective promotion of men involvement in the protection of women’s reproductive rights needs a baseline data of husbands’ knowledge, involvement, and related factors. However, little is known about the issue in Ethiopia at large and in Bahir Dar city specifically. Therefore, this study aimed to assess husband’s knowledge on women SRHRs, husbands’ involvement when their wives practice their SRHRs and to identify factors associated with husbands’ involvement in Bahir Dar City, Northwest Ethiopia.

## Methods

### Study, setting, design, and period

Community-based cross-sectional study design was employed in Bahir Dar city, Ethiopia. Bahir Dar is the capital of Amhara administrative regional state, located 563 km away from Addis Ababa. According to the Bahir Dar City Municipality office 2019\2020 report, Bahir Dar city has 6 sub-cities and 26 kebeles with a total population of 312,410; of these, 145,579 are male individuals. According to Bahir Dar sub-cities’ administrative 2021/22 report, 99, 238 married male households are living in the city. As per Bahir Dar City administrative health department report, there are 23 governmental health facilities and four private hospitals and few more private clinics. From 23 governmental health facilities: three hospitals, ten health centers, and ten health posts. The health facilities provide various reproductive health services based on the capacity of health facilities in terms of available human resources and equipment including essential drugs. The services are including counseling and provision of contraception, STIs including HIV/AIDS testing and management, safe motherhood service (antenatal, intrapartum and postnatal), cervical cancer screening and treatment, infertility screening and treatment, safe abortion and post-abortion care services, and effective referral linkage. The data were collected from March 20 to April 5, 2023.

### Source and study population

All men who were married and had at least one child in Bahir Dar city were the source population, while all men who fulfilled the above criteria and lived in randomly selected sub-cities were the study population.

### Inclusion criteria

Those husbands, who were married, lived in the city at least for the past 6 months preceding the survey and had at least one child included in this study.

### Sample size determination and sampling procedure

The sample size (n) for this study was calculated using single Kothari formula

$$n=\frac{Z^{2}*pq*N}{e^{2}\left(N-1\right)+Z^{2}*pq}\text{,}$$

and this formula used for finite population, considered; proportion (p) 40.1% which was husbands’ level of involvement in Harar, East Ethiopia (14), 95% level of confidence (1.96), and 5% margin of error.

$$n=\frac{3.841*0.401\left(1-0.401\right)*39227}{0.0025\;\left(39227-1\right)+3.841*0.401\left(1-0.401\right)}$$

$$n=\frac{36191.001}{98.9876}=366$$

By considering a 10% non-response rate, the final sample size for this study was 403.

**Figure 1:**
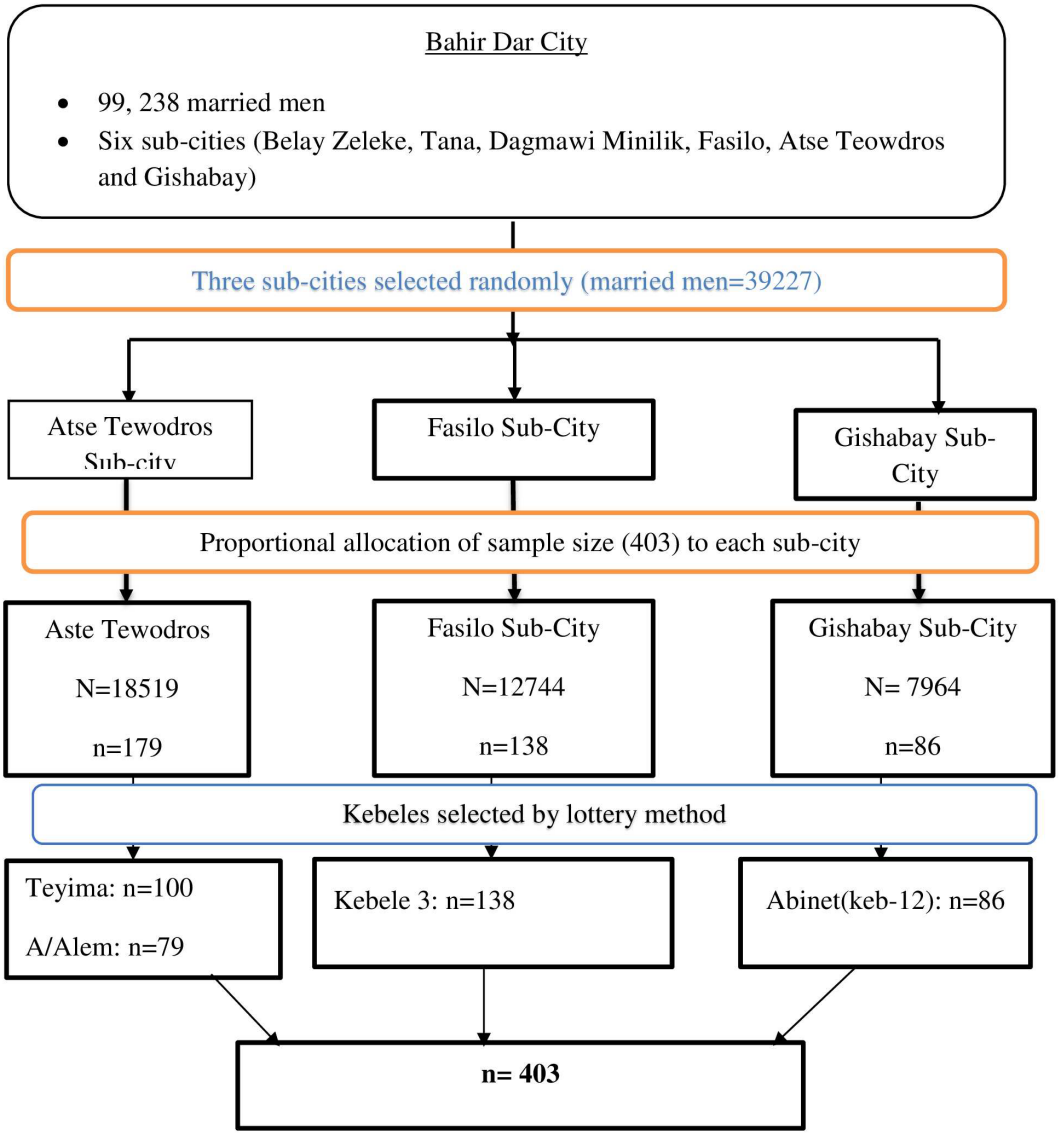
Schematic presentation of sampling procedure in Bahir Dar city, 20,223.

Regarding sampling procedure, Bahir Dar City has six sub-cities, and three sub-cities were selected by lottery method. The total sample size was allocated proportionally to each sub-city based on the total married men living in each sub-city (Figure 1).

### Sampling techniques

A multi-stage sampling technique was used to select sub-cities from six sub-cities. In the first stage, three sub-cities were selected from Bahir Dar city using a lottery method by considering the rule of thumb of 30% coverage of representative of the study population. In the second stage; four kebeles were selected from three sub-cities using lottery method. The total calculated sample husbands were allocated proportionally to each kebeles based on the total number of households. In the third stage, total numbers of married households were taken from each kebele administration using simple random sampling method until the allocated sample size was reached. When each selected household had more than one respondent (study unit), one person was selected by the lottery method at the time of data collection. In non-response after the repeated visit (two times), the individual was considered non-response.

### Study variables

Husband involvement in their wives’ SRHRs was the outcome variable; while *socio-demographic and economic-related variables* (age, income, husband’s father raised up, educational level, occupation, religion, and types of marriage); *source of* i*nformation-related* var*iables* (exposure to media, training/education on SRHR, open discussion with family members and friends); *and Knowledge on SRHRs* were independent variables.

### Data collection tool and procedure

The data collection tool for this study was adapted from prior published international literatures (9, 14, 17, 19–22). The primary source of information/data for this study was husbands. The data were collected using translated to local language (Amharic) and pretested structured questionnaire through face-to-face interview. One-day training was given for two male data collectors and one supervisor who had data collection and supervision experiences. The data collection procedure was done in private rooms/areas since the topic was sensitive.

### Definition of terms

#### Reproductive right

It is the right of an individual to decide and choose about reproduction free of discrimination, coercion, and violence (23).

#### Reproductive health

It is a state of complete physical, mental, and social wellbeing not merely the absence of diseases and infirmity in all matters of relating to the reproductive system and its functions and process (24, 25).

#### Sexual health

It is a positive approach to human sexuality, and the purpose of sexual healthcare should be to enhance life and personal relations, counseling and care related to reproduction and sexually transmitted diseases (25).

#### Sexual rights

Embrace human rights that are already recognized in national laws, international human rights documents, and other consensus documents. These include the right of all persons, to be free of coercion, discrimination, and violence, to live to the highest attainable standard of health in relation to sexuality, including access to sexual and reproductive healthcare services; seek, receive, and impart information in relation to sexuality; sexuality education; respect for bodily integrity; choice of partner; decide to be sexually active or not; consensual sexual relations; consensual marriage; decide whether or not, and when to have children; and pursue a satisfying, safe, and pleasurable sexual life (25).

#### Sexuality

It is a central aspect of being human throughout life and encompasses sex, gender identities and roles, sexual orientation, eroticism, pleasure, intimacy, and reproduction. Sexuality is experienced and expressed in thoughts, fantasies, desires, beliefs, attitudes, values, behaviors, practices, roles, and relationships. It can be influenced by the interaction of biological, psychological, social, economic, political, cultural, ethical, legal, historical, religious, and spiritual factors (25).

#### Sexual and reproductive health

It is a state of physical, mental, emotional, and social wellbeing about sexuality and reproduction (24).

#### Sexual and reproductive health rights

These are the rights of sexual and reproductive health, which includes the right to freely control and responsibly decide on matters related to sexuality, including sexual and reproductive decision-making and the ability to seek sexual and reproductive healthcare that is free of coercion, discrimination, violence, the number of babies, and spacing (6).

### Operational definition

#### Discussion with family members and friends

Refers to a discussion on sexual and reproductive health issues with husband/male involvement and initiated by himself or friends or any family members including women, reproductive aged daughters, or/and sons,

### Measurements

#### Knowledge of husband

It was measured using 22 standard reproductive right questions, each scored 1 or 2 (1 = no and 2 = yes). Those who scored above the total mean (>1.73) value of knowledge-measuring questions were considered knowledgeable; otherwise, they were considered not knowledgeable (14, 26).

#### Husband’s involvement

It is husband’s activity or support in his wife’s SRHRs when the wife practices her SRHRs at home and workplace. The outcome variable was coded as 1 = No, 2 = Yes of husband’s involvement after computing thirteen (13) involvement Likert scale questions were used to assess husbands’ level of involvement when their wives’ practice their SRHRs and each item scored 1 to 5(1 = never, 2 = rarely, 3 = sometimes, 4 = often, and 5 = always). The study participants’ minimum and highest possible score were 13 and 65, respectively (14, 26, 27). Husbands’ who scored above median (>2) of involvement-measuring questions were categorized as having good involvement (Yes = 2) in their wives’ sexual and reproductive rights.

### Data analysis and presentation

The data were coded and entered Epi-data version 4.6 and exported to the Statistical Package for Social Sciences (SPSS) version 25 software for analysis. Descriptive data analysis (frequency and percentage) was used to extract values from the raw data. Both bivariate and multivariate logistic regression models were done to estimate the association between independent variables and outcome variable (husbands’ involvement). Independent variables with *p*-value of less than 0.25 in the bi-variable analysis were entered into the multivariable logistic regression analysis and *p*-value <0.05 was cutoff point to declare statistical significance in the final regression model. Backward elimination (Backward LR) was applied in multivariable analysis to control confounding variables. Hosmer–Lemeshow model test with p-value >0.05 showed fitted model with this study p-value of 0.121. Finally, adjusted odd ratio (AOR) with 95% CI was used to show the strength of association between the outcome and independent variables.

### Data quality control

The quality of data was assured by different mechanisms. The questionnaire was first prepared in English and translated into Amharic (local language) and retranslated to English to check its consistence. Then, the questionnaire was pretested among 5% (21 participants) of the sample size in kebel-7, Bahir Dar before the start of actual data collection, and we made minor modifications to its content. One-day training was provided for data collectors and supervisors. The data collection process was supervised closely, and the completeness of each questionnaire was checked by the investigator and supervisor on a daily basis. The content and technical aspect of the tool was reviewed by experts. Internal consistency of Likert scale category of variables was checked using the Cronbach alpha value of 0.849.

## Results

### Socio-demographic and economic characters of participants

A total of 391 out of 403 husbands participated in this study with a response rate of 97.02%. In total, 241 (61.1%) of the study participants’ father grew up in rural area, and 161 (41.2%) of study participants’ fathers were unable to read and write due to their educational status. The mean age of the study participants was 41.77 years with (SD ± 11.68), and 115 (29.4%) of the study participants were with age range of 40–49 years. More than half (59.8%) of the study participants lived together with their wives between 5 and 25 years. Nearly, one-fourth (24%) of the study participants were first degree by educational level, and more than two-thirds (67%) of the study participants were orthodox Christian religion followers. Regarding occupation, 183(46.8%) of the study participants were engaged in personal business activity, and more than one-thirds (37.3%) of the study participants had arranging marriage by type. Lastly, six in ten (59.3%) of the study participants’ monthly income was > or = 4,600 Ethiopian birr (Table 1).

**Table 1:** Socio-demographic and economic-related factors of the study participants and their extended families in Bahir Dar city, Ethiopia, 2023 (*N* = 391).

Variable	Response	Frequency	Percent (%)
Place husband’s father grew up	Urban	150	38.4
	Rural	241	61.6
Husband’s father educational level	Unable to read and right	161	41.2
	Able to read right	134	34.3
	Primary school (1–8)	54	13.8
	Secondary school (9–12)	28	7.2
	Certificate	4	1.0
	First degree and above	10	2.6
Age of the study participant	Less than 30 years old	66	16.9
	30–39 years old	114	29.2
	40–49 years od	115	29.4
	> or = 50 years old	96	24.6
Live together with wife in years	Less than 5 years	120	30.7
	From 5 to 25 years	234	59.8
	> or = 26 years	37	9.5
Educational level	Unable to read and write	30	7.7
	Able to read and write	85	21.7
	Elementary school (1–8)	54	13.8
	High school (9–12)	52	13.3
	Certificate (diploma)	14	3.6
	First degree	94	24.0
	Second degree and above	62	15.9
Religion	Orthodox	262	67.0
	Muslim	98	25.1
	Protestant	31	7.9
Type of marriage	Arranged	146	37.3
	Love	245	62.7
Occupation	Farmer	22	5.6
	Private business	183	46.8
	Governmental employee	164	41.9
	Student	2	0.5
	Daily laborer	14	3.6
	Others^*^	6	1.5
Monthly income in birr	Less than 4,600 birrs	159	40.7
	> or = 4,600 birr	232	59.3

^*^ = non-governmental organizations (NGOs) and contractors.

### Source of information and husbands’ knowledge on SRHRs-related characteristics

Nearly, three-fourths (73.4%) of the study participants did not access any training/education opportunity about reproductive health, and almost two-thirds (63.9%) of the study participants did not discuss with their friends and/or family members about SRHRs.

Regarding husbands’ knowledge on SRHRs, 198 (50.6%) of them had good knowledge after computing knowledge assessment questions. More than three-forth (76.7%) of the respondents replied that they aware about women SRHRs without verified the components. Among those who aware about women SRHRs, health institutions (72.3%) were the leading source of information followed by 182(60.7%) mainstream media (radio and Television). Of the total 370 husbands who knew that their wives have the right to use their choice of family planning methods, 281 (75.9%) knew injectable type of family planning method. Almost one-fifth (23.8%) of the study participants did not know that their wives have the right to receive screening and treatment of STIs including HIV/AIDS and urinary tract infections (UTIs). More than three-fourth (77.2%) of the study participants knew that they and their wives have equal reproductive health rights and 177 (45.3%) of the husbands did not know about their wives have the right to enjoy and control their sexual and reproductive life. Almost half (48.8%) of the study participants did not know that having sex whenever they want irrespective of their wives’ will be punishable by the law. More than half (57.8%) of the husbands did not know that forced marriage and/or abusive sexual language is punishable by law. Finally, three-fourth (73.1%) of the study participants did not aware about safe abortion with certain conditions is legal by law in Ethiopia (Table 2).

**Table 2:** Source of information and knowledge of study participants on SRHRs, Bahir Dar city, Ethiopia, 2023 (*N* = 391).

Variable	Response	Frequency	Percent
Aware about wives’ SRHRs	No	91	23.3
	Yes	300	76.7
Source of information (*N* = 300) #multiple answer was possible	Health institution	217	72.3
	Leaflet/brochures	62	20.7
	News Paper	51	17
	Television and/or Radio	182	60.7
	Friends	37	12.3
	Workplace	44	14.7
	Religious institution	19	6.5
	Others^*^	13	4.3
Husband knows that his wife can decide when to get marriage previously	No	136	34.8
	Yes	255	65.2
Husband knows that his wife can decide when to get pregnancy	No	105	26.9
	Yes	286	73.1
Husband knows that his wife can decide the number of her children	No	90	23.0
	Yes	301	77.0
Husband knows that his wife can decide the space between children	No	39	10.0
	Yes	352	90.0
Husband knows that his wife has the right to use any contraception	No	21	5.4
	Yes	370	94.6
Type/s of family planning methods husbands know (*N* = 370) #Multiple answer was possible	Pill	275	74.3
	Injectable	281	75.9
	Norplant	155	41.9
	Male sterilization	40	10.8
	IUD	63	17
	Natural method	66	17.8
	Condom	59	15.9
Husband knows that his wife has the right to get prenatal healthcare services	No	45	11.5
	Yes	346	88.5
Husband knows that his wife has the right to get safe delivery care services/institutional delivery	No	30	7.7
	Yes	361	92.3
Husband knows that his wife has the right to receive post-natal healthcare services	No	48	12.3
	Yes	343	87.7
Husband knows that his wife has the right to get screening and treatment of STIs including HIV/AIDS and UTI	No	93	23.8
	Yes	298	76.2
Husband knows that he and his wife have equal reproductive health rights	No	89	22.8
	Yes	302	77.2
Husband knows that his wife has the right of her reproductive health issues must be kept confidential/secret	No	62	15.9
	Yes	329	84.1
Husband knows that his wife has the right to enjoy and control their sexual and reproductive life	No	177	45.3
	Yes	214	54.7
Husband knows that having sex whenever he wants irrespective of his wife’s will is punishable by the law	No	191	48.8
	Yes	200	51.2
Husband knows that share childcare is an obligation	No	22	5.6
	Yes	369	94.4
Husband knows that he and his wife have equal rights in decision-making	No	127	32.5
	Yes	264	67.5
Husband knows that sometimes/occasionally punish/hit/insult his wife is acceptable (physically, psychologically or sexually) NB: validated at the time of interview	No	144	36.8
	Yes	247	63.2
Husband knows that using forced marriage and/or abusive sexual language is punishable by law	No	226	57.8
	Yes	165	42.2
Husband knows that safe abortion with certain conditions is legal by law in Ethiopia	No	286	73.1
	Yes	105	26.9
Husband knows that his wife has the right to get abortion care services and post-abortion care with certain conditions	No	174	44.5
	Yes	217	55.5
Husband knows that his wife has the right for screening and treatment of infertility	No	73	18.7
	Yes	318	81.3
Husband knows that his wife has the right for cervical cancer screening and treatment	No	59	15.1
	Yes	332	84.9

Others*; community health workers, neighbors and family members, and IUD; Intrauterine Device.

### Husbands’ involvement in wives SRHRs

**Figure 2:**
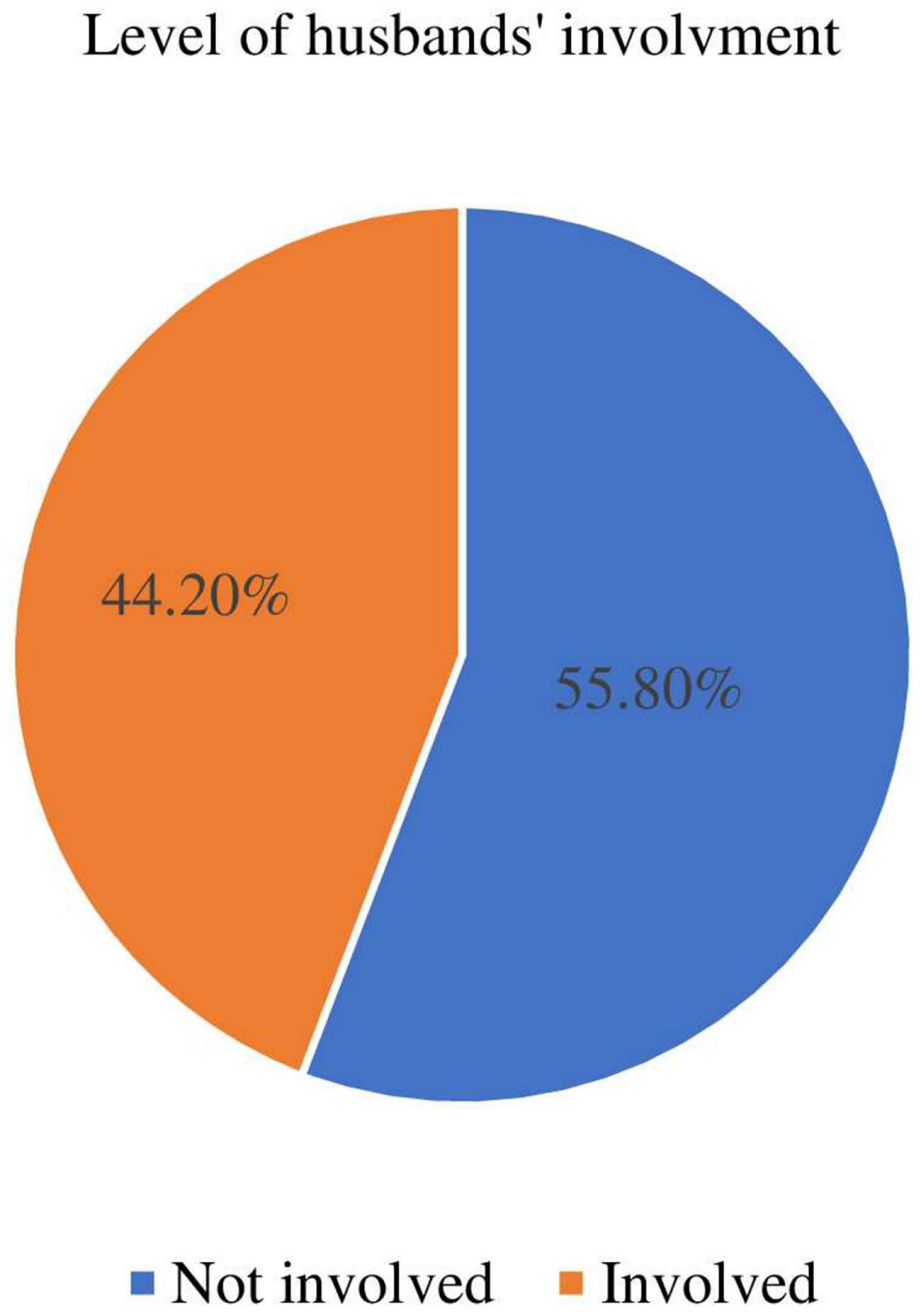
Husbands’ level of involvement in their wives SRHRs in Bahir Dar City, Ethiopia, 2023 (*N* = 391).

Of the overall respondents, 173(44.2%) were involved in their wives SRHRs (Figure 2).

From Likert scale questions, a total of 192 (49.1%) study participants were not reminding where and when family planning services are needed for their wives. More than half (57.6%) of the participants never had sex whenever they need irrespective of wives’ interest. Half (50.6%) of the husbands were not encouraging their wives to get reproductive health-related information/education. Regarding antenatal visit, more than one-thirds (38.6%) of husbands had never gone with their wives for ANC follow-up; of these, 60 (39.7%) mentioned that havening ANC follow-up is females’ job as a reason for not involved. In total, 118 (30.8%) of the husbands did not go with their wives for institutional childbirth service ever and 91 (23.3%) were not involved in PNC follow-up. A promising finding in this study was that 138 (35.3%) husbands often supported their wives when they breastfed. Sixty-two (15.9%) of the husbands did not involve in their wives screening/testing for STIs, including HIV/AIDS and of these, 26 (41.9%) respondents mentioned busy for other duties as a reason for not being involved. More than half (51.9%) of the husbands never discussed openly with their wives about sexual desire and when to have sex and only 92 (23.5%) study participants were always ready to go with their wives if they need cervical cancer screening and treatment (Table 3).

**Table 3:** Husbands’ involvement by each Likert scale measurement items in Bahir Dar City, Northwest Ethiopia, 2022/23(*N* = 391).

Variable	Response	Frequency	Percent
Husbands knew that they remind their wives when and where they need FP	Never	192	49.1
	Rarely	45	11.5
	Sometimes	57	14.6
	Often	71	18.2
	Always	26	6.6
Participants had sex whenever they want irrespective of their wife’s interest	Never	226	57.8
	Rarely	32	8.2
	Sometimes	51	13.0
	Often	65	16.6
	Always	17	4.3
Participants encouraged wives to acquire RH-related information/education	Never	198	50.6
	Rarely	76	19.4
	Sometimes	35	9.0
	Often	42	10.7
	Always	40	10.2
Husbands went for ANC with their wives	Never	151	38.6
	Rarely	99	25.3
	Sometimes	62	15.9
	Often	41	10.5
	Always	38	9.7
The reason for never for safer ANC (*N* = 151)	Busy with other duties	49	32.5
	It is their (females) job	60	39.7
	I do not know whether it’s important to her	23	15.2
	Cultures and norms not supported	19	12.6
Husbands went with their wives to get institutional delivery (birth) service	Never	118	30.2
	Rarely	87	22.3
	Sometimes	45	11.5
	Often	71	18.2
	Always	70	17.9
The reason for husbands Never gone with their wives to get institutional delivery and service (*N* = 118)	Busy with other duties	30	25.4
	It is their (females) job	55	46.6
	I do not know whether it’s important to her	21	17.8
	Cultures and norms not supported	12	10.2
Respondents went with their wives to have safe PNC	Never	91	23.3
	Rarely	133	34.0
	Sometimes	69	17.6
	Often	57	14.6
	Always	41	10.5
The reason for husbands never visited health facilities for safe PNC with their wives (*N* = 91)	Busy with other duties	17	18.7
	It is their (females) job	14	15.4
	I do not know whether it’s important to her	42	46.2
	Cultures and norms not supported	18	19.8
Husbands supported when wives breastfeeding	Never	13	3.3
	Rarely	34	8.7
	Sometimes	108	27.6
	Often	138	35.3
	Always	98	25.1
Husband involved in screening for STIs, including HIV/AIDS	Never	62	15.9
	Rarely	103	26.3
	Sometimes	40	10.2
	Often	99	25.3
	Always	87	22.3
The reason for husbands were Never involved in screening for STIs, including HIV/AIDS (*N* = 62)	Busy with other duties	26	41.9
	It is their (females) job	11	17.7
	I do not know whether it’s important to her	14	22.6
	Cultures and norms not supported	11	17.7
Respondents need to encourage their wives to use safe abortion care service	Never	168	43.0
	Rarely	41	10.5
	Sometimes	60	15.3
	Often	67	17.1
	Always	55	14.1
Husbands supported their wives before abortion and post-abortion care	Never	133	34.0
	Rarely	72	18.4
	Sometimes	71	18.2
	Often	47	12.0
	Always	68	17.4
Respondents need to help/assist their wives to aware that she had equal reproductive rights like your	Never	130	33.2
	Rarely	66	16.9
	Sometimes	36	9.2
	Often	80	20.5
	Always	79	20.2
Husbands openly discussed with their wives about sexual desire and when to have sex	Never	203	51.9
	Rarely	69	17.6
	Sometimes	53	13.6
	Often	24	6.1
	Always	42	10.7
Husbands went with their wives if they need cervical cancer screening and treatment	Never	65	16.6
	Rarely	57	14.6
	Sometimes	113	28.9
	Often	64	16.4
	Always	92	23.5

HIV/AIDS, human immunodeficiency virus/Acquired immunodeficiency syndrome, F/P, family planning, ANC, antenatal care, PNC, postnatal care and STIs, Sexual transmitted infections.

### Factors affecting husbands’ involvement in their wives SRHRs

Binary (bivariate and multivariate) logistic regression was run to identify factors associated with the outcome variable. In bivariate logistic regression, six variables were associated with *p*-value less than 0.25. After controlling confounding factors, five variables were significantly associated with outcome variable in multivariate logistic regression at p-value <0.05. Those who had a monthly income of <4,600 Ethiopian birrs were 9.25 times more likely to involve in their wives SRHRs compared to those who had > or = 4600birr monthly income with [AOR = 9.25; 95% CI (4.17–20.5)]. Study participants who had second degree and above by educational level were 8.8 times more likely to involve in their wives SRHRs compared with those who were unable to read and write husbands [AOR = 8.8; 95% CI (2.04–38)]. The odds of study participants involvement in their wives SRHRs were almost 6 times higher among those who accessed training/education opportunity about reproductive health compared to those who did not with [AOR = 5.99; 95% CI (2.71–13.2)]. The study participants who had open discussion about SRHRs with family members and/or friends were 1.92 times more likely to involve in their wives SRHRs compared with those who had not get discussion with [AOR = 1.92; 95% CI (1.01–3.63)]. Finally, the odds of involvement were 7.9 times higher among those who had good knowledge on SRHRs issue compared with their counterparts with [AOR = 7.9; 95% CI (4.28–14.4)] (Table 4).

**Table 4:** Regression table shows the association between independent and dependent variables, Bahir Dar City, 2023.

Variables	Husbands were involved		COR; 95% CI	AOR;95% CI	*p*-value
	No (%)	Yes (%)			
**Husbands’ father level of education**					
Unable to read and right	97 (44.5)	64 (37)	1.00	1.00	1.00
Able to read right	77 (35.3)	57 (32.9)	1.122 (0.74–1.79)	0.51 (0.26–1.03)	0.06
Primary school (1–8)	32 (14.7)	22 (12.7)	1.042 (0.56–1.95)	1.16 (0.47–2.88)	0.74
Secondary school (9–12)	12 (5.5)	16 (9.2)	2.021 (0.89–4.55)	0.35 (0.08–1.48)	0.15
Certificate	0(0)	4(2.3)	2,448,454… (000–…)	101,405,204…	0.99…
first degree and above	0(0)	10(5.8)	2,448,454… (0.00–…)	400,648,289…	0.99…
**Monthly income**					
Less than 4,600 birr	76 (34.9)	83 (48)	1.72 (1.15–2.59)	9.25 (4.2–20.5)*	0.00
> or = 4,600 birr	142 (65)	90 (52)	1.00	1.00	1.00
**Husband educational level**					
Unable to read and write	24 (11)	6 (3.5)	1.00	1.00	1.00
Able to read and write	50 (22.9)	35 (20.2)	2.8 (1.04–7.56)	1.49 (0.39–5.77)	0.56
Elementary school (1–8)	37 (17)	17 (9.8)	1.84 (0.64–5.32)	1.12 (0.3–4.06)	0.87
High school (9–12)	34 (15.6)	18 (10.4)	2.12 (0.73–6.12)	3.68 (0.84–16.2)	0.084
Certificate (diploma)	7 (3.2)	7 (4)	4.0 (1.01–15.9)	5.1 (0.5–11.8)	0.22
First degree	31 (14.2)	63 (36.4)	8.13 (3.01–21.9)	13.1 (0.8–52.1)	0.09
Second degree and above	35 (16.1)	27 (15.6)	3.09 (1.11–8.61)	8.81 (2.04–38)*	0.004
**Access training/education opportunity about reproductive health**					
No	183 (83.9)	104 (60.1)	1.00	1.00	1.00
Yes	35 (16.1)	69 (39.9)	3.47 (2.16–5.57)	5.99 (2.7–13.2)*	0.00
**Discuss about SRHRs with family members and/or friends**					
No	156 (71.6)	94 (54.3)	1.00	1.00	1.00
Yes	62 (28.4)	79 (45.7)	2.12 (1.39–3.22)	1.92 (1.01–3.6)*	0.046
**Husbands level of knowledge**					
Poor	146 (67)	47 (27.2)	1.00	1.00	1.00
Good	72 (33)	126 (72.8)	5.44 (3.51–8.42)	7.94 (4.3–14.4)*	0.00

*; significantly associated with the outcome variable at *p*-value < 0.05 and 1.00; references.

## Discussion

This study was conducted using a quantitative study approach that intended to assess husbands’ knowledge and involvement and factors influencing husbands’ involvement in their wives sexual and reproductive health rights in Bahir Dar city, Northwest Ethiopia. The level of husbands’ involvement and knowledge was 44.2% (95% CI; 39.3–49.1%) and 50.6% (95%CI, 48.07–53.13%), respectively. Husbands’ level of education, monthly income, access of training/education opportunity about reproductive health, had open discussion about SRHRs with family members and/or friends, and husbands’ level of knowledge were the factors significantly associated with husbands’ involvement.

The level of knowledge in this study is in line with the study conducted in Harar, Eastern, Ethiopia (48.3%) (14). These similarities might be due to short time gap, similar study population (urban resident), and the study participants were married husbands in both studies. However, this study is lower than the study conducted in Wolaita Sodo, Ethiopia (54.5%) (9), Gondar, Ethiopia (57.7%) (28), Adet Tana Haik (59.6%) (29), Debre Markos, Ethiopia (67%) (30), and Ghana (80%) (13). The difference might be due to variation of study population socio-demographic profile, gender of study participants, and study design. The study conducted in Gondar, Wolaita Sodo, Debre Markos, and Adet was among university and college students who aimed to assess their knowledge on female SRHRs. College/university students usually participated or members in reproductive health clubs, which may give more opportunity to understand girls/women’s SRHRs. Moreover, students may have SRHRs information exposure from their class courses, life skill trainings and communication/discussion with their friends/peers. The difference with Ghana might be due to study design and study population, both sexes of adolescent students were involved in the study. On other hand, the level of knowledge in this study is higher than the study conducted in Sri Lanka (24.4%) (20), Nepal (37%) (23), India (9.1%) (26), Nigeria (45.2%) (17), and shire town, Northern Ethiopia (47.1%) (31). This might be because of cultural, socio-demographic profile of the study population, study area, and year of study differences.

Level of husbands’ involvement in this study is in line with the study conducted in Bangladesh (40%) (32), Bale zone, Southern Ethiopia (41.4%) (16), Afar, Ethiopia (42.2%) (21), and Harar, Eastern Ethiopia (40.1%) (14). The plausible reason for the similarity might be due to the same socio-economic background of study population, and all studies were conducted in recent years. However, the level of involvement in this study is lower than studies conducted in Nepal (57.6%) (33), Debre Markos, Northwest Ethiopia (60%) (34) and Tanzania (69%) (35). The probable reasons for the difference might be sociocultural and economic status, husbands’ level of education, time/year of study, the accessibility and implementation of SRHRs components. The study conducted in Debre Markos; Northwest Ethiopia was not evaluated all components of SRHRs rather specific on family planning involvement, while the study conducted in Nepal and Tanzania are better in implementation of laws and regulation and done on single component of SRHRs which was antenatal follow-up. Probably religious norms are also the barriers for low involvement of husbands in SRHRs. In this study, more than two-thirds (67%) of the study participants were orthodox Christian religion followers. One study in Afar, Ethiopia revealed that religious leader husbands were 30% less likely to involve during their wife’s family planning service uptake compared with husbands who were not religious leaders (21). Beside this, poor health system and not having a health policy that encourages the practice of SRHRs in Ethiopia may be another reason for low husbands’ involvement. On the other side, this study is higher than the study conducted in Ghana (20%) (19) and Harar, Eastern Ethiopia (19.7%) (36). The probable reason for the discrepancy might be due to cultural differences, quality and availability of healthcare service infrastructures, sample size, and component of SRHRs were assessed. The study conducted in Ghana was on husbands’ involvement in postnatal care service utilization which is one component of SRHRs. One study highlighted that cultural standards were identified as a barrier for male involvement (37).

The regression analysis showed that husbands who had <4,600 Ethiopian birr monthly income were more likely to involve in their SRHRs compared with those who had monthly income of > or = 4,600 birr. This study agrees with qualitative study conducted in Nepal (38) that showed; *“mostly males are involved in income generation, they do not have enough time and no concerns about wives SRHRs*.” The likely reason for the association might be that husbands with better monthly income could be busy with different duties/responsibilities and give their time for generating money over helping their wives.

Education is an important instrument/strategy to know about something and to change the mind set of human behavior. Husbands who had advanced educational level were 8.8 times more likely to be involved in their wives SRHRs when compared to their counterparts. This finding is supported with the study conducted in Nepal (33) and Tanzania (35). One study reported that adequate information on SRHRs directly impacts the involvement of sexual and reproductive rights, changing attitude, and overcoming some sociocultural-related barriers (21).

Those husbands who accessed training/education opportunities about reproductive health were 6 times more likely to be involved in their wives SRHRs compared with their counterparts. This finding is supported with study conducted in Myanmar, Nepal and Malawi (39, 40). The plausible reason for the association could be the more the husbands have knowledge on SRHRs would have the better involvement. The study participants who had open discussion about SRHRs with family members and/or friends were 1.92 times more likely to involve in their wives SRHRs compared with who did not have discussion, which agrees with studies conducted in Harar, Ethiopia (14), Bangladesh (32), and India (41). The probable reason could be those who had an open discussion with their friends and/or family about SRHRs may boost their knowledge and then yield good involvement in their wives SRHRs. One study proved that an open discussion between husbands and wives resulted good knowledge and involvement of the husbands on women reproductive issues (28). Another study also proved that poor communication between husband and their female partners was associated with poor male involvement (34). Finally, in this study, the odds of involvement were 7.9 times higher among those who had good knowledge on SRHRs issue compared to their counterparts. This finding is agreed with the study conducted in Afar, Ethiopia (21), Nigeria (17), Nepal (38) and Myanmar (39). It is obvious that when the level of husbands’ knowledge increased, the possibility their involvement in their wives SRHRs could be also increased.

### Implication of the study

National and International conventions recommend husband involvement is necessary in women’s SRHRs; however, more efforts are needed to implement different international conventions. At community level; community leaders should create awareness about traditional, cultural practice norms and barriers affecting husbands’ involvement in wives SRHRs. Finally, legal affairs and women federation must adopt policies and strategies that sustained the presence of husband involvement in SRHRs through education/trainings. The government needs to develop a male involvement strategy to encourage and support men and boys to take responsibility of their sexual and reproductive behavior and to abstain from all forms of discrimination against women and girls. Husbands’ knowledge and involvement in their wives SRHRs increase their awareness, acceptance and support to their partner’s needs, choices, and rights. Men participation in women SRHRs encourages prompt care in wives’ safe motherhood services. An engaged father who feels responsible for and behaves responsibly toward his child, is emotionally engaged and physically accessible, involved in childcare, rearing provides material and support to sustain the children’s needs.

### Limitations of the study

This study has some limitations. The study setting was urban; therefore, the finding could not be generalizable for rural husbands. Moreover, the study did not show a real cause-and-effect relationship due to the nature of the study design, which was a cross-sectional study. Finally, due to the nature of the topic sensitivity, this study might introduce reporting bias despite all efforts to minimize it.

## Conclusion

In this study, the level of husbands’ knowledge and involvement in their wives’ SRHRs were low. Access training/education about sexual health, husbands’ advance educational level, having good knowledge about SRHRs, low monthly income (<4,600 birr), and open discussion with family members and friends about SRHRs were significantly associated with husbands’ involvement when their wives exercise SRHRs. Therefore, responsible concerned bodies need to work on the strategies that help to improve men involvement and knowledge, and tackle the above-mentioned factors influencing their involvement.

## Data availability statement

The raw data supporting the conclusions of this article will be made available by the authors, without undue reservation.

## Ethics statement

The ethical approval letter for this study was obtained from the Ethical Review Committee of the Bahir Dar University (reference number: GDS-25/15). Permission letters were received from each selected sub-cities. The study was conducted in accordance with local legislation and institutional requirements. The participants provided their written informed consent to participate in this study.

## Author contributions

MJ: Formal analysis, Investigation, Methodology, Writing – original draft, Writing – review & editing. MZ: Methodology, Software, Supervision, Validation, Writing – original draft, Writing – review & editing. BR: Data curation, Formal analysis, Methodology, Supervision, Validation, Writing – original draft, Writing – review & editing.
